# [Retracted] MicroRNA-132 induces temozolomide resistance and promotes the formation of cancer stem cell phenotypes by targeting tumor suppressor candidate 3 in glioblastoma

**DOI:** 10.3892/ijmm.2025.5517

**Published:** 2025-03-10

**Authors:** Zhen-Xiu Cheng, Wen-Bo Yin, Zhong-Yu Wang

Int J Mol Med 40: 1307-1314, 2017; DOI: 10.3892/ijmm.2017.3124

Following the publication of this paper, it was drawn to the Editors' attention by a concerned reader that the clonogenic agar assay data shown in Fig. 5C on p. 1312 were strikingly similar to data appearing in different form in another article written by different authors at different research institutes, which had already been published in the journal *Oncotarget* prior to the submission of this paper to *International Journal of Molecular Medicine*. In view of the fact that the abovementioned data had already apparently been published previously, the Editor of *International Journal of Molecular Medicine* has decided that this paper should be retracted from the Journal. The authors were asked for an explanation to account for these concerns, but the Editorial Office did not receive a reply. The Editor apologizes to the readership for any inconvenience caused.

